# Insights Into Health-Related Quality of Life of Kidney Transplant Recipients: A Narrative Review of Associated Factors

**DOI:** 10.1016/j.xkme.2025.100986

**Published:** 2025-02-25

**Authors:** Tim J. Knobbe, Daan Kremer, Ute Bültmann, Coby Annema, Gerjan Navis, Stefan P. Berger, Stephan J.L. Bakker, Yvette Meuleman

**Affiliations:** 1 Division of Nephrology, Department of Internal Medicine, University Medical Center Groningen, University of Groningen, Groningen, The Netherlands; 2 Community and Occupational Medicine, Department of Health Sciences, University Medical Center Groningen, University of Groningen, Groningen, The Netherlands; 3 Section of Nursing Science, Department of Health Sciences, University Medical Center Groningen, University of Groningen, Groningen, The Netherlands; 4 Department of Clinical Epidemiology, Leiden University Medical Center, Leiden, Netherlands

## Abstract

Life expectancy and graft survival continue to improve after transplantation. However, improved posttransplant clinical outcomes do not necessarily translate into improved health-related quality of life (HRQoL). Therefore, there is an increased focus on HRQoL in kidney transplant recipients (KTRs). The HRQoL of KTRs is worse than that of the general population, but interventions that improve HRQoL in KTRs are scarce, and health care professionals in nephrology care do not routinely address HRQoL. To improve HRQoL, it is essential to understand which factors play a role in HRQoL and to pinpoint areas for intervention. This narrative review maps the concept of HRQoL within the KTR population and provides a comprehensive overview of factors associated with posttransplant HRQoL. The results are structured using an easy-to-understand conceptual model of HRQoL, which is instrumental for understanding how HRQoL is constituted of many clinical and nonclinical factors. We conclude that symptom burden among KTRs is high, which is likely a key driver of the limited HRQoL in this population. Moreover, myriad other clinical and nonclinical factors are associated with HRQoL, but the majority of the evidence is observational.

## Introduction

Health-related quality of life (HRQoL) after kidney transplantation is notably better than before kidney transplantation and surpasses HRQoL of patients treated by dialysis.^1^ However, posttransplant HRQoL is substantially worse than that of the general population.^1^ Clinical outcomes after kidney transplantation improve,^2^ yet this improvement does not necessarily translate to enhanced HRQoL, which is concerning because maintaining or improving HRQoL has been identified as one of the most important outcomes by patients with chronic kidney disease.^3^^,^^4^ Additionally, HRQoL is consistently associated with relevant clinical outcomes, including mortality, in patients with chronic kidney disease,^5^ as well as with graft failure and mortality in kidney transplant recipients (KTRs).^6^^,^^7^ Therefore, there is an increasing focus on identifying factors affecting HRQoL of KTRs.

HRQoL reflects an individual’s *appraisal* of their health and the limitations they experience in life because of their health. It differs from the broader concept ‘quality of life’, which also takes into account patients’ spiritual, political, cultural, and economic circumstances.^8^^,^^9^ HRQoL is further explained in Box 1.^10^

Box 1 **The concept of health-related quality of life**

Health-related quality of life (HRQoL) encompasses the appraisal (ie, value judgment) of how an individual perceives and values the impact of health-related limitations on their overall quality of life, shaped by any limitations they experience in life because of health problems. Limitations may be encountered in multiple domains of health, including:

• Physical functioning: the capacity for engaging in physical activities, such as walking, climbing stairs, or participating in sports.
• Mental functioning: overall psychological well-being, including thinking and information processing.
• Emotional functioning: the ability to manage emotions, cope with stress, and experience feelings such as joy and sadness in a balanced way.
• Social functioning: the capability to form and maintain satisfying relationships, to participate in social activities, and to interact within a community.

HRQoL is distinct from functioning/functional status, because HRQoL *also* encompasses an individual’s appraisal (ie, value judgment) of their functioning. Thus, it takes into account the impact of limitations on their life and the extent to which an individual finds these limitations bothersome or distressing.^8^

Importantly, HRQoL encompasses not only the negative impact of health but also the positive aspects of an individual’s well-being, such as satisfaction in relation to their health.

To date, an overview of factors associated with HRQoL among KTRs is lacking, which makes identification of areas for intervention challenging. In this narrative review, we provide an overview of factors associated with HRQoL among KTRs. To this end, we searched PubMed using the search terms: “(health-related) quality of life” or “(HR)QoL” and “kidney/renal transplantation” or “kidney/renal transplant recipients.” Relevant articles and references of included articles were screened for studies missed by the searching strategy. Only publications written in English were included, and no restrictions were applied regarding publication date or study design. It is important to acknowledge that this review is limited to a narrative synthesis, and that, in this emerging field, a large proportion of the evidence is based on (cross-sectional) observational studies. For clarification purposes, the evidence regarding associations with HRQoL presented in this narrative review is derived from cross-sectional studies using observational data unless otherwise specified (eg, using evidence from longitudinal observational studies, randomized controlled trials and other types of interventional studies, and systematic reviews and meta-analyses).

## Factors Associated With HRQoL Among KTRs

**Figure 1:**
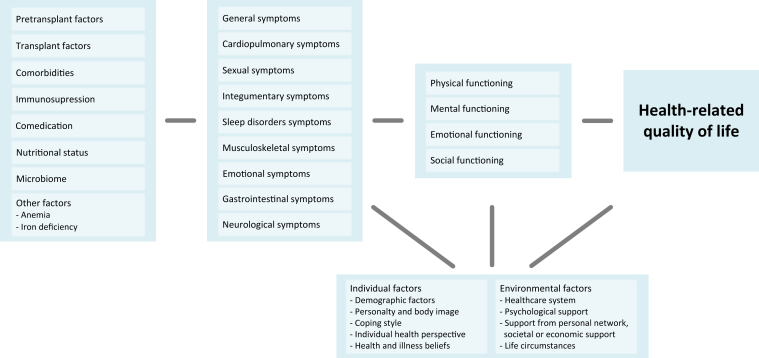
Factors associated with health-related quality of life among kidney transplant recipients, organized in a manner inspired by Wilson and Cleary’s^8^ conceptual model of health-related quality of life. From left to right: biological and physiological factors, symptom status, physical functioning, and health-related quality of life. Below, characteristics of the individual and environment.

HRQoL after kidney transplantation is influenced by many factors. These factors of influence may be structured following the conceptual model of HRQoL by Wilson and Cleary^8^ that is broadly applied in health psychology and is instrumental for understanding how HRQoL is affected by or associated with many clinical and nonclinical factors.^11^^,^^12^ Our version of the model, adapted to the KTR context, is presented in Fig 1.

The model explicates multiple levels of influence, including the following: (1) biological and physiological variables, (2) symptom status, (3) functional status, and (4) characteristics of the individual and environment. Although each of these concepts is unique, they are closely related. Biological and physiological impairments are often expressed as symptoms. Symptoms, in turn, may result in functional limitations. The experience of these symptoms and the extent to which they affect functional status are influenced by individual and environmental factors that determine how symptoms and functional limitations are perceived, managed, and eventually affect HRQoL.^8^

Each level will be described in detail in the corresponding section. Although these health aspects are closely related—sometimes even combined into a single health questionnaire—it is crucial to distinguish between them. By merging them into a single patient-reported health aspect, we risk losing valuable insights into how different health-related factors shape broader health concepts such as HRQoL.

### Biological and Physiological Variables

Biological and physiological variables are fundamental determinants of health and encompass the function of cells, organs, and organ systems.^8^ Among these biological and physiological variables, we found the following factors associated with posttransplant HRQoL.

#### Pretransplant Factors

Pretransplant factors, such as a Lui comorbidity index score of 7 or greater and a longer dialysis vintage, have been associated with a decline in HRQoL post-transplantation in a longitudinal study.^13^ However, despite the clinical preference for preemptive transplantation, a meta-analysis including only 3 studies observed similar HRQoL in preemptively transplanted patients and those who underwent dialysis beforehand. It should be noted that this meta-analysis did not take into account the time patients spent being treated with dialysis and that the level of evidence was considered low given the high risk of bias.^14^ Small uncontrolled interventional studies showed that prehabilitation before kidney transplantation improves fatigue and physical fitness parameters,^15^ and participants’ feedback suggested that prehabilitation helped them to be more physically and mentally prepared for transplantation.^16^ However, it remains unknown whether prehabilitation effects also translate into improved HRQoL post-transplantation, and large randomized controlled trials assessing the effects on HRQoL are lacking.

#### Transplant Factors

Transplant factors, such as shorter ischemia time, lower donor age, living donor transplant, and lower number of HLA mismatches have been associated with better HRQoL,^17^^,^^18^ likely through their downstream effects on graft outcomes, such as kidney function. Indeed, longitudinal studies have shown that poor kidney function is associated with a decline in HRQoL.^6^^,^^19^ Additionally, donor-derived infections can have a negative impact on recipients’ HRQoL.^20^^,^^21^ For example, it has been shown that allocation of kidneys from cytomegalovirus-negative donors to cytomegalovirus-negative recipients was associated with superior quality-adjusted life years.^21^ Furthermore, a transplant rejection is associated with worse HRQoL.^22^^,^^23^ Additionally, transplantation can have psychological effects such as feelings of guilt, worry about the graft, fear of rejection, stress, or anxiety.^24^^,^^25^

#### Comorbid Conditions

KTRs are at high risk of having and developing comorbid conditions, such as cardiovascular diseases, malignancy, infections, and posttransplant diabetes, which are negatively associated with HRQoL.^26^^,^^27^ Additionally, these comorbid conditions frequently require hospital admissions, which is also associated with worse HRQoL.^26^^,^^28^

#### Immunosuppression

Immunosuppression, the cornerstone of posttransplant care, plays a significant role in posttransplant symptoms. Induction therapy generally consists of an interleukin-2 receptor antagonist.^29^ Although interleukin-2 receptor antagonists are preferred for low-risk patients, a randomized controlled trial demonstrated that KTRs aged ≥50 years were significantly more likely to improve in HRQoL when treated with an induction therapy consisting of lymphocyte-depleting agent rabbit antithymocyte globulin.^29^^,^^30^

After induction therapy, most KTRs receive lifelong triple therapy, encompassing calcineurin inhibitors, proliferation inhibitors, and steroids.^29^ Among calcineurin inhibitors, tacrolimus is often favored over cyclosporin for superior graft outcomes.^29^ However, both medications have distinct side effects, prompting occasional switches to mitigate symptom burden. Belatacept offers an alternative: 2 randomized controlled trials showed that HRQoL was significantly better in belatacept users than in cyclosporin users.^17^ However, no studies have compared their outcomes with tacrolimus.^29^^,^^31^ Additionally, the higher treatment burden of belatacept, because of its intravenous administration, cannot be overlooked. Two randomized controlled trials investigated the effect of reducing or discontinuing calcineurin inhibitor use on HRQoL and found no HRQoL difference with reduced calcineurin inhibitor doses after daclizumab induction.^28^^,^^32^ However, among mTOR inhibitor users, discontinuation of the calcineurin inhibitor cyclosporin had a beneficial effect on HRQoL.^32^ Notably, a regimen consisting of an mTOR inhibitor with a reduced dosage of calcineurin inhibitor was shown to be effective and safe.^33^ Furthermore, although steroids are often a part of post-transplantation regimens,^29^ steroid-free protocols do exist, which can reduce patient burden of comorbid conditions and symptoms.^34^ However, the effects of steroid-free regimens on patient and graft outcomes remain controversial, rendering their use debatable.^34^

#### Comedication

Medications such as proton pump inhibitors have been associated with worse HRQoL, possibly because of their impact on gastrointestinal dysbiosis and micronutrient uptake.^35^ Although warranting additional investigation, reevaluating proton pump inhibitor prescriptions could be an option for HRQoL enhancement, especially considering the widespread yet often unnecessary proton pump inhibitor usage among KTRs.^35^^,^^36^ It is important to note that, although not yet investigated, other medications frequently prescribed to KTRs (eg, statins, β-blockers, and α-blockers), may also be associated with worse HRQoL via their side effect profiles.

#### Nutritional Status

Malnutrition, inadequate diet, and insufficient protein intake are prevalent in KTRs and are associated with worse HRQoL.^27^^,^^37^, ^38^, ^39^ However, 2 randomized controlled trials did not show any beneficial effects of a dietary intervention on HRQoL.^40^^,^^41^

#### Microbiome

The gut plays a role in regulating mood, behavior, and overall well-being via the gut–brain axis.^42^ Many KTRs suffer from gut dysbiosis (ie, overgrowth of pathogens at the expense of commensal bacteria), which is associated with worse HRQoL.^43^ For instance, the presence of several butyrate-producing bacteria, especially *Faecalibacterium prausnitzii* (which is typically abundant in healthy individuals but less common in those with a disease), has been linked to better HRQoL.^43^

#### Other Factors

Posttransplant anemia, affecting 20%-51% of KTRs, is associated with worse HRQoL in KTRs.^44^^,^^45^ Two randomized controlled trials demonstrated that correcting anemia with epoetin-β was safe and improves HRQoL in KTRs.^45^^,^^46^ Similarly, iron deficiency, present in up to 47% of KTRs, has been associated with worse HRQoL, fatigue, concentration problems, and depressive symptoms, regardless of coexisting anemia.^47^^,^^48^ The association is possibly because of the essential role of iron in cardiovascular, muscle, and brain function.^47^ Furthermore, airflow limitation, a condition characterized by reduced lung compliance and obstruction, is seen in 25% of KTRs and is associated with worse HRQoL and risk of fatigue.^49^ However, this association appears to be explained by the impact of muscle mass and strength on HRQoL.^50^

### Symptom Status

In line with the conceptual model, symptom status is influenced by biological and physiological variables.^8^ The prevalence and etiology of the most occurring symptoms among KTRs and their associations with HRQoL are addressed in the following sections.

#### General

Fatigue, affecting a striking 40%-50% of KTRs, is considered one of the most burdensome symptoms and is a key factor associated with worse HRQoL, affecting patients’ functioning in almost all health domains, as demonstrated in a systematic review and meta-analysis.^51^ It has been suggested that both physiological and psychological factors play a role.^51^^,^^52^ Hence, managing fatigue is crucial in KTRs but requires a comprehensive approach using strategies to treat not only physiological causes but *also* psychological (contributing) factors.

#### Cardiopulmonary

Approximately half of KTRs experience reduced cardiorespiratory fitness and a tendency toward a sedentary lifestyle, which consequently can negatively affect patients’ functional status and HRQoL.^17^^,^^53^, ^54^, ^55^ A meta-analysis of randomized controlled trials showed that exercise interventions can improve the HRQoL of KTRs.^56^^,^^57^ The authors concluded that caution is needed when interpreting the results of existing studies because of their relatively small sample sizes, high risk of bias, and lack of postintervention follow-up. However, recently, 2 large randomized controlled trials showed that an exercise intervention improved HRQoL,^41^^,^^58^ with a digital intervention also being proven cost effective.^58^ Finally, physical inactivity and palpitations have been associated with worse HRQoL.^6^^,^^59^

#### Sexual

Sexual symptoms including reduced sexual desire and orgasmic and erectile dysfunction occur in 38%-66% of KTRs.^54^^,^^60^, ^61^, ^62^ The etiology of sexual symptoms is multifactorial, encompassing physical, psychological (eg, anxiety and negative body image), and pharmacologic factors (particularly immunosuppressive drugs).^60^, ^61^, ^62^ These factors may therefore be potential therapeutic targets. An overview of drugs affecting sexual function and potential management strategies has been published previously.^61^ Importantly, sexual dysfunction is associated with worse HRQoL.^6^^,^^60^^,^^62^^,^^63^ An uncontrolled intervention study observed that sildenafil improved erectile dysfunction, sexual life, partner relationships, and overall life satisfaction in male KTRs.^64^ Additionally, menstrual problems have been associated with worse HRQoL.^6^

#### Integumentary

Mucocutaneous symptoms are frequently reported by KTRs, largely because of immunosuppressive therapy.^54^^,^^65^, ^66^, ^67^, ^68^ Manifestations vary but primarily concern neoplasms and infections such as warts, herpes, and mycoses.^65^, ^66^, ^67^ Other inflammatory or cosmetic manifestations, such as hair loss/overgrowth, acne, urticaria, dry skin, and bruises, are also prevalent.^65^^,^^66^ Mucocutaneous manifestations often lead to distress among KTRs are associated with worse HRQoL.^67^^,^^69^

#### Sleep Problems

KTRs often experience sleep problems,^54^^,^^70^^,^^71^ with approximately half of female KTRs and one-third of male KTRs reporting poor sleep.^71^ Anxiety appears to be a major determinant.^71^ Poor sleep is strongly associated with fatigue and worse societal participation.^71^ Additionally, a systematic review and a recent, large observational study demonstrated its association with HRQoL.^70^^,^^71^ It is important to identify any causes of sleep disturbances, such as nocturia, because they can show potential targets for intervention.^70^^,^^71^

#### Musculoskeletal

Muscle weakness is commonly reported among KTRs. Muscle function is required for almost all daily activities and participation in society, which likely explains its strong association with HRQoL, as demonstrated in cross-sectional and longitudinal studies.^17^^,^^19^^,^^72^ This association appears to be nonlinear, with a strong association of muscle mass or strength with HRQoL below a certain point and a much weaker or absent association beyond this point.^50^ Finally, muscle cramps also occur and have been negatively associated with HRQoL.^6^^,^^54^

#### Emotional

KTRs face numerous emotional stressors, both pretransplant (eg, coping with kidney disease, dialysis dependency, waiting list anxiety, and loss of work) and posttransplant (eg, fear of rejection and potential return to dialysis). Such stressors can trigger psychological issues. Indeed, studies report prevalences of approximately 26% for depressive symptoms or depression, 7%-25% for anxiety, and 0.4%-21% for posttraumatic stress disorder^24^^,^^54^^,^^73^, ^74^, ^75^, ^76^—all of which are associated with worse HRQoL.^6^^,^^17^^,^^19^^,^^24^ However, posttransplant emotional problems often remain unaddressed during consultations.^77^ When identified, physicians should consider seeking advice or referring the patient to an expert such as a social worker, psychologist, or psychiatrist, depending on the situation at hand. Addressing psychological issues can also foster posttraumatic growth, such as improved self-esteem or ability to put things into perspective, which has been associated with better HRQoL of KTRs.^24^

#### Gastrointestinal

Of KTRs, 88%-92% experience gastrointestinal symptoms, with reported prevalences of 57%-83% for indigestion, 45%-69% for abdominal pain, 37%-58% for constipation, 53%-54% for diarrhea, and 45%-47% for reflux, among others.^54^^,^^78^^,^^79^ The high prevalence and severity of these symptoms may have significant implications for HRQoL, more than clinicians often estimate.^6^^,^^17^^,^^78^, ^79^, ^80^ A small, uncontrolled interventional study showed that KTRs experiencing severe mycophenolate mofetil-related gastrointestinal side effects had fewer symptoms and better HRQoL after switching from mycophenolate mofetil to enteric-coated mycophenolate sodium.^81^ A randomized controlled trial showed no effects of prebiotic supplementation in KTRs on HRQoL during a 7-week follow-up, although gastrointestinal symptoms decreased.^82^

#### Neurological

Tremors, affecting 40%-69% of KTRs, and headache, affecting 32%-44% of KTRs, are frequently reported as distressful and hindering daily activities.^6^^,^^54^^,^^63^^,^^83^^,^^84^ (Poly)neuropathy may cause pain, paresthesia, and numbness,^85^ and restless legs, affecting 5%-19% of KTRs, may cause sleep problems.^54^^,^^86^ Furthermore, approximately 16%-38% of KTRs exhibit (mild) cognitive impairments.^87^^,^^88^ Moreover, approximately 25% of KTRs experience impaired hand dexterity and skill in performing precise and coordinated hand movements, which has been associated with a reduced societal participation.^89^ Consequently, these symptoms can limit patients in their daily life and have been negatively associated with HRQoL.^63^^,^^83^^,^^86^^,^^87^^,^^89^

### Functional Status

Symptom status impacts functional status, which refers to an individual’s ability to perform tasks.^8^ This encompasses activities of daily living, self-care, societal participation, family and social interactions, and (maintaining) independence. KTRs experience functional limitations, particularly in physical domains, which likely have a direct and significant impact on their HRQoL, as depicted in the example in Box 2.^89^ Furthermore, KTRs with functional limitations may become unemployed, resulting in a loss of daily routine, social contact, income, and poorer HRQoL.^90^ Although employed KTRs report to function well at work, likely because of adaptations they have made, posttransplant symptoms have been associated with worse work functioning.^91^

Box 2 **Example: impact of tremor on health-related quality of life**

Experiencing tremor can lead to problems with eating or drinking by making it difficult to bring food or liquids to the mouth, which can affect nutritional status and the ability to go out for dinner. This may also cause problems with hygiene, which can affect independence. Additionally, tremors can cause issues with writing or performing precise tasks, which can influence the ability to engage in hobbies or work.^83^ Following the conceptual model, a symptom such as tremor can adversely affect an individual’s functioning in multiple life domains and consequently negatively influence health-related quality of life.

### Characteristics of the Individual and Environment

As depicted in Fig 1, individual and environmental characteristics influence symptom status, functional status and HRQoL.^8^

#### Individual Factors

Individual factors affect perceptions and responses to symptoms or situations.^11^ Factors such as being male, being younger, having a higher educational level, cohabiting, positive body image, and helpful personality traits are associated with better HRQoL among KTRs.^6^^,^^19^^,^^26^^,^^92^, ^93^, ^94^, ^95^ Furthermore, KTRs with avoidance and maladaptive coping strategies (eg, excessive sleeping) report worse HRQoL than those with active coping strategies (eg, seeking social support or problem solving).^96^ Therefore, fostering adequate coping strategies could positively affect HRQoL.^97^ Additionally, although not yet confirmed in KTRs, patient preferences and values may influence perceptions of health status. This means that what one individual considers a burdensome symptom or functional limitation, others might view as a minor inconvenience. Such preferences and values may also be shaped by one’s (kidney) disease history, experiences with dialysis, and expectations about life post-transplantation. Finally, when patients are faced with a health threat such as a diagnosis or symptoms, it evokes cognitive and emotional perceptions (ie, how serious and controllable is the situation?). These illness perceptions affect how patients cope with disease and perceive HRQoL.^97^^,^^98^

#### Environmental Factors

Environmental factors, such as emotional and practical support from personal networks, societal or economic support, housing situation, internet access, and the health care system (eg, accessibility and chronic illness management), can affect HRQoL.^11^^,^^26^ Indeed, 3 randomized controlled trials demonstrated that patient education or intensive guidance post-transplantation had beneficial effects on HRQoL.^99^, ^100^, ^101^ Notably, one of these care programs was found to be cost effective.^101^

## Recommendations for Clinical Practice

Our review provides a framework that can help health care professionals better understand HRQoL after kidney transplantation. This framework can, alongside results from routinely collected patient-reported outcome measures (PROMs, measuring HRQoL and symptom burden), enhance clinical practice by educating both health care professionals and patients about potential factors associated with posttransplant HRQoL. To equip health care professionals with more concrete treatment options to improve HRQoL in KTRs, there is an urgent need for longitudinal and randomized interventional studies. Currently, most evidence is derived from cross-sectional and observational studies, necessitating considerable caution regarding causality and suggestions for strategies in clinical practice. However, this narrative synthesis provides a clear overview of potential targets of intervention that may enhance HRQoL. Following the conceptual model and in line with the systematic review of Kugler et al,^102^ we conclude that addressing symptom burden may be a very important step toward improving HRQoL in KTRs. Moreover, given the multifaceted nature of HRQoL and the broad range of potential factors that appears to be associated with HRQoL, clinicians should—besides treatment options given by themselves—also consider paramedical treatment options and seek advice of other experts such as dermatologists, urologists, sexologists, sleep therapists, occupational therapists, dieticians, and psychologists. Importantly, HRQoL should be systematically monitored over time (also to evaluate treatment effects), and results should be discussed with patients as part of routine nephrology care. It is advisable to administer the PROM before the visit with the health care professional, allowing for an in-person discussion of the results during the consultation. Please see Box 3 for a selection of the available and suitable questionnaires to measure HRQoL in this population.^103^ Conducting a HRQoL assessment before transplantation is also encouraged to allow for comparison with post-transplantation HRQoL, and a decrease in frequency of HRQoL assessment may be considered with increasing time posttransplant (eg, from once per month to a frequency of once per year).

Box 3 **Health-related quality of life measures among kidney transplant recipients**

Various reliable and validated questionnaires (ie, PROMs [patient-reported outcome measures]) to measure health-related quality of life (HRQoL) exist. The most commonly used questionnaires to assess HRQoL among kidney transplant recipients are the 36-Item Short-Form Health Survey^104^ (SF-36, a generic questionnaire to measure HRQoL; also available as shortened version, the SF-12) and the Kidney Disease Quality of Life^105^ (KDQOL, a kidney disease-specific questionnaire).^1^^,^^10^ Recently, the Patient-Reported Outcomes Measurement Information System^104^ (PROMIS) has gained increasing attention as generic questionnaire to measure HRQoL, available as a static Short-Form and in an adaptive format (using computerized adaptive testing) in which follow-up questions are selected from an item bank based on answers to the already administered questions. Finally, 2 other disease-specific questionnaires for kidney transplant recipients are the Renal Transplant Quality of Life Questionnaire Second Version^106^ (ReTransQoL V2) and the Chronic Kidney Disease-specific Quality-Of-Life Computerized Adaptive Test (CKD-QOL-CAT).^10^^,^^107^

## Recommendations for Research

In the field of kidney transplantation research, there is a notable shift toward focusing on HRQoL rather than solely on mortality and graft failure—a trend that likely continues in the upcoming decades. It is therefore of the utmost importance to increase our knowledge on this matter, bring together the expertise of medical and social sciences, conduct rigorous (longitudinal) studies, and perform pragmatic trials. We propose that clinical trials should always include HRQoL as an outcome. However, it should be noted that this can increase trial costs (eg, coordinator time to ensure data are adequately collected). Importantly, the use of a PROM is advised by both the US Food and Drug Administration and the European Medicines Agency.^108^^,^^109^ Additionally, there is a high need for specific interventional trials aimed at improving HRQoL, in which the factors provided in Box 4 may be considered as potential targets for intervention.

Box 4 **Factors associated with a worse health-related quality of life in kidney transplant recipients**

**Biological and physiological variables**

• Worse allograft function because of factors related to the surgery, donor, and recipient
• Donor-derived infections
• Impact of kidney transplant rejection
• Comorbid conditions
• Hospital admissions
• Side effects of (immunosuppressive) medication
• Inadequate diet, potentially leading to malnutrition
• Gut dysbiosis
• Anemia, iron deficiency, and airflow limitation

**Symptoms status**

• Fatigue
• Reduced cardiorespiratory fitness, reduced sport activity, and palpitations
• Sexual symptoms (eg, sexual desire, orgasmic and erectile dysfunction) and menstrual problems
• Mucocutaneous symptoms (eg, neoplasms and infections)
• Sleep problems
• Muscle weakness and cramps
• Depressive symptoms and depression
• Anxiety
• Posttraumatic stress disorder
• Gastrointestinal symptoms (eg, indigestion, abdominal pain, and chronic diarrhea)
• Tremor, (poly)neuropathy, impaired hand dexterity, and headache
• Cognitive impairments

**Functional status**

• Functional limitations in daily life
• Loss of work (and consequently a loss of income, daily routine, etc)

**Characteristics of the individual and environment**

• Demographic factors (eg, higher age, being female, and lower educational level)
• Unhelpful personality traits and negative body image
• Maladaptive coping strategies (eg, avoidance)
• Unrealistic expectations of post-transplantation outcomes
• Lack of support from personal network, lack of societal or economic support
• Unhelpful illness perceptions
• Housing situation (eg, not having access to an elevator)
• Impaired or suboptimal health care system
• No internet access

## Conclusions

We conclude that symptom burden among KTRs is high, which is likely a key driver of the limited HRQoL in this population. Moreover, myriad other clinical and nonclinical factors are associated with HRQoL, but the majority of the evidence is derived from cross-sectional and observational studies. Rigorous longitudinal observational and intervention studies are needed to substantiate the evidence base and to explore whether interventions targeted toward the identified factors may improve HRQoL in KTRs.
